# Supplementary material for: Integration of Multiomic Data to Characterize the Influence of Milk Fat Composition on Cantal-Type Cheese Microbiota
Source: Microorganisms. 2022 Feb 1;10(2):334. doi: 10.3390/microorganisms10020334 (PMC8879305; doi:10.3390/microorganisms10020334)
Supplement: Supplementary file 1 [file microorganisms-10-00334-s001.zip › microorganisms-1537608-supplementary.pdf]

Table S1. α-diversity indices for bacterial communities; significant p-values between P- and M-derived samples are written in italic bold; for each index, values not sharing the same superscript letter by line are significantly different.

		Number of observed ASVs						Chao1					
		Milk	Core D3	Core D150	Rind D30	Rind D90	Rind D150	Milk	Core D3	Core D150	Rind D30	Rind D90	Rind D150
Raw milk	Pasture	40 ± 4^{abc}	46 ± 14^{ac}	35 ± 16^{c}	52 ± 17^{abc}	66 ± 26^{b}	69 ± 7^{ab}	40 ± 5^{a}	48 ± 16^{a}	38 ± 18^{a}	51 ± 17^{a}	72 ± 27^{a}	70 ± 8^{a}
	Maize	39 ± 7^{abc}	38 ± 8^{ac}	35 ± 3^{c}	50 ± 9^{abc}	69 ± 16^{b}	66 ± 7^{ab}	41 ± 8^{ab}	39 ± 7^{a}	36 ± 4^{a}	53 ± 9^{ab}	77 ± 28^{b}	67 ± 7^{ab}
	p-value	0.322	0.409	0.956	0.388	0.253	0.803	0.531	0.337	0.874	0.367	0.320	0.773

		Shannon						Simpson					
		Milk	Core D3	Core D150	Rind D30	Rind D90	Rind D150	Milk	Core D3	Core D150	Rind D30	Rind D90	Rind D150
Raw milk	Pasture	1.27 ± 0.35^{ab}	$0,67 \pm 0,18^{ab}$	0.20 ± 0.06^{b}	0.57 ± 0.40^{ab}	1.15 ± 0.64^{ab}	1.78 ± 0.36^{a}	0.54 ± 0.18^{a}	0.18 ± 0.06^{a}	0.06 ± 0.01^{a}	0.18 ± 0.14^{a}	0.39 ± 0.22^{a}	0.61 ± 0.12^{a}
	Maize	0.95 ± 0.42^{ab}	0.69 ± 0.18^{ab}	0.40 ± 0.28^{a}	0.69 ± 0.68^{ab}	1.33 ± 0.41^{b}	1.63 ± 0.46^{b}	0.37 ± 0.24^{a}	0.29 ± 0.34^{a}	0.13 ± 0.09^{a}	0.23 ± 0.08^{a}	0.46 ± 0.15^{a}	0.56 ± 0.19^{a}
	p-value	0.294	0.792	0.097	0.433	0.315	0.686	0.280	0.636	***0.033***	0.418	0.298	0.745

		Number of observed ASVs						Chao1					
		Milk	Core D3	Core D150	Rind D30	Rind D90	Rind D150	Milk	Core D3	Core D150	Rind D30	Rind D90	Rind D150
Pasteurized milk	Pasture		33 ± 7^{a}	22 ± 4^{b}	14 ± 3^{b}	40 ± 7^{a}	48 ± 4^{a}		33 ± 7^{a}	16 ± 2^{ac}	15 ± 3^{b}	39 ± 11^{ac}	50 ± 5^{a}
	Maize		30 ± 7^{abc}	15 ± 2^{c}	15 ± 5^{ac}	34 ± 10^{b}	45 ± 4^{b}		31 ± 7^{abc}	26 ± 5^{c}	16 ± 5^{ac}	40 ± 7^{b}	45 ± 4^{b}
	p-value		0.502	<0.001	0.297	0.484	0.526		0.553	***0.019***	0.474	0.527	0.347

		Shannon						Simpson					
		Milk	Core D3	Core D150	Rind D30	Rind D90	Rind D150		Core D3	Core D150	Rind D30	Rind D90	Rind D150
Pasteurized milk	Pasture		0.70 ± 0.26^{abc}	0.30 ± 0.13^{ac}	0.50 ± 0.12^{a}	1.01 ± 0.20^{b}	1.28 ± 0.33^{b}		0.24 ± 0.09^{ab}	0.17 ± 0.06^{b}	0.20 ± 0.06^{ab}	0.36 ± 0.06^{ab}	0.45 ± 0.12^{a}
	Maize		0.50 ± 0.10^{ac}	0.24 ± 0.01^{c}	0.31 ± 0.13^{ac}	0.81 ± 0.27^{a}	1.56 ± 0.29^{b}		0.17 ± 0.03^{ac}	0.09 ± 0.01^{c}	0.13 ± 0.07^{ac}	0.30 ± 0.08^{a}	0.58 ± 0.08^{b}
	p-value		0.677	0.473	0.299	0.419	0.437		0.695	0.522	0.456	0.541	0.286

Table S2. α-diversity indices for fungal communities; significant p-values between P- and M-derived samples are written in italic bold; for each index, values not sharing the same superscript letter by line are significantly different.

		Number of observed ASVs			Chao1			Shannon			Simpson		
		**Rind D30**	**Rind D90**	**Rind D150**	**Rind D30**	**Rind D90**	**Rind D150**	**Rind D30**	**Rind D90**	**Rind D150**	**Rind D30**	**Rind D90**	**Rind D150**
**Raw milk**	**Pasture**	16 ± 1^{a}	13 ± 2^{a}	14 ± 4^{a}	16 ± 1^{a}	13 ± 2^{a}	14 ± 4^{a}	1.08 ± 0.11^{a}	0.87 ± 0.13^{a}	0.91 ± 0.20^{a}	0.47 ± 0.06^{a}	0.47 ± 0.07^{a}	0.41 ± 0.10^{a}
	**Maize**	14 ± 2^{a}	14 ± 1^{a}	12 ± 4^{a}	15 ± 2^{a}	14 ± 1^{a}	12 ± 4^{a}	1.08 ± 0.11a	0.97 ± 0.25^{a}	0.34 ± 0.09^{b}	0.62 ± 0.05^{a}	0.49 ± 0.12^{a}	0.13 ± 0.04^{b}
	**p-value**	0.741	0.933	0.741	0.676	0.933	0.779	0.067	0.816	***0.043***	0.080	0.691	***0.048***

		Number of observed ASVs			Chao1			Shannon			Simpson		
		**Rind D30**	**Rind D90**	**Rind D150**	**Rind D30**	**Rind D90**	**Rind D150**	**Rind D30**	**Rind D90**	**Rind D150**	**Rind D30**	**Rind D90**	**Rind D150**
**Pasteurized milk**	**Pasture**	12 ± 2^{a}	13 ± 4^{a}	15 ± 3^{a}	12 ± 2^{a}	13 ± 4^{a}	15 ± 3^{a}	1.04 ± 0.31^{a}	1.13 ± 0.33^{a}	0.72 ± 0.42^{a}	0.46 ± 0.14^{a}	0.56 ± 0.14^{a}	0.29 ± 0.18^{a}
	**Maize**	10 ± 1^{a}	12 ± 2^{a}	10 ± 2^{a}	10 ± 1^{a}	12 ± 2^{a}	10 ± 2^{a}	1.13 ± 0.26^{a}	1.09 ± 0.29^{a}	0.26 ± 0.05^{b}	0.52 ± 0.14^{a}	0.54 ± 0.13^{a}	0.09 ± 0.02^{b}
	**p-value**	***0.031***	0.742	0.111	***0.031***	0.657	0.111	0.466	0.966	0.169	0.547	0.936	0.171

Table S3. Relative abundance of differential bacterial taxa found differentially abundant between P- and M-derived cheese rinds.

	**Taxon**	**Raw milk cheeses**						**Pasteurized milk cheeses**					
		**Maize**			**Pasture**			**Maize**			**Pasture**		
		**D30**	**D90**	**D150**	**D30**	**D90**	**D150**	**D30**	**D90**	**D150**	**D30**	**D90**	**D150**
**Dominant**	*Brevibacterium* spp.	0.0353	0.1557	0.1702	0.0268	0.1184	0.2602	0.0831	0.2225	0.0253	0.0624	0.1236	0.0367
	Enterobacter spp.	0.0073	0.0037	0.0020	0.01144	0.0077	0.0016	0.0009	0.0021	< 0.0001	0.0214	0.01358	0.0029
	Brachybacterium spp.	0.0007	0.0380	0.0489	0.0011	0.0580	0.0652	0.0149	0.0530	0.0055	0.0101	0.0498	0.0152
	Yaniella halotolerans	0.0000	0.0139	0.0273	0.0001	0.03454	0.03122	0.0004	0.0025	0.0004	0.0007	0.0007	0.0013
	Staphylococcus equorum	0.0008	0.0057	0.0111	0.0002	0.0073	0.0093	0.0012	0.0070	0.0059	0.0011	0.0037	0.0068
	Hafnia alvei	0.0068	0.0069	0.0036	0.0107	0.0103	0.0031	0.0018	0.0043	0.0006	0.01370	0.0119	0.0019
	Lactococcus spp.	0.9299	0.7521	0.7055	0.9198	0.7283	0.5887	0.8924	0.6995	0.9594	0.8841	0.7847	0.9267
**Sub-dominant**	*Nocardiopsis* sp.	0.0000	0.0004	0.0000	0.0000	< 0.0001	0.0030	< 0.0001	0.0007	0.0001	0.0000	0.0024	0.0015
	Stackebrandtia nassauensis	0.0000	0.0000	0.0000	0.0000	0.0000	0.0016	0.0000	< 0.0001	0.0000	0.0000	< 0.0001	< 0.0001
	Ruania aldibiflava	0.0000	0.0000	0.0000	0.0000	0.0000	0.0014	0.0000	< 0.0001	0.0000	0.0001	0.0001	0.0000
	Corynebacterium sp.	0.0000	0.0000	< 0.0001	0.0000	< 0.0001	0.0016	0.0001	< 0.0001	0.0000	< 0.0001	0.0000	0.0000
	Dietzia timorensis	0.0000	0.0002	0.0003	< 0.0001	0.0004	0.0040	0.0017	0.0007	< 0.0001	0.0005	0.0006	0.0001
	Dietzia psychralcaliphila	0.0001	0.0013	0.0017	0.0004	0.0008	0.0085	0.0018	0.0024	0.0004	0.0022	0.0020	0.0013
	Lactobacillus sp.	0.0006	0.0030	0.0064	0.0008	0.0042	0.0030	0.0000	0.0000	0.0004	0.0000	0.0000	0.0000
**Rare**	*Salinicoccus* sp.	0.0000	< 0.0001	0.0003	0.0000	< 0.0001	0.0003	< 0.0001	0.0002	0.0000	0.0000	0.0001	< 0.0001
	Chryseobacterium haifense	0.0006	0.0005	0.0004	0.0004	0.0004	< 0.0001	0.0000	0.0000	0.0000	0.0000	0.0000	0.0000
	Bartonella coopersplainsensis	< 0.0001	0.0002	0.0004	0.0002	0.0003	0.0003	< 0.0001	0.0007	0.0003	0.0001	0.0011	0.0004
	Acinetobacter johnsonii	0.0002	< 0.0001	0.0000	< 0.0001	0.0001	< 0.0001	< 0.0001	0.0000	0.0000	0.0000	0.0000	0.0000
	Jeotgalicoccus psychrophilus	< 0.0001	0.0003	0.0007	< 0.0001	0.0006	0.000	0.0002	0.0002	< 0.0001	0.0000	< 0.0001	0.0000
	Acinetobacter berezinae	0.0017	0.0010	0.0006	0.0017	0.0013	0.0009	< 0.0001	0.0001	0.0000	0.0007	0.0007	0.0002
	Methylobacterium sp.	0.0000	0.0000	0.0000	< 0.0001	< 0.0001	0.0000	0.0000	< 0.0001	< 0.0001	0.0001	0.0000	0.0000
	Flavobacterium sp.	0.0000	0.0000	0.0000	0.0000	0.0000	0.0000	0.0000	0.0000	0.0000	< 0.0001	0.0005	0.0000
	Acinetobacter sp.	0.0005	0.0006	0.0003	0.0005	0.0007	0.0002	0.0000	0.0002	0.0000	0.0002	0.0004	0.0002
	Jeotgalicoccus saudimassiliensis	0.0000	0.0000	0.0000	0.0000	< 0.0001	0.0000	0.0000	< 0.0001	< 0.0001	0.0000	0.0000	0.0000

	Janibacter limosus	0.0000	< 0.0001	< 0.0001	0.0000	0.0002	0.0007	0.0002	< 0.0001	< 0.0001	0.0001	0.0000	0.0000

Table S4. Relative abundance of differential fungal taxa found differentially abundant between P- and M-derived cheese rinds.

	Taxon	Raw milk cheeses						Pasteurized milk cheeses					
		**Maize**			**Pasture**			**Maize**			**Pasture**		
		**D30**	**D90**	**D150**	**D30**	**D90**	**D150**	**D30**	**D90**	**D150**	**D30**	**D90**	**D150**
**Dominant taxa**	Microascaceae	0.0029	0.0032	0.0274	0.0004	0.0028	0.0981	0.0004	0.0067	0.0063	0.0093	0.0078	0.0770
	Sporendonema casei	0.0489	0.6748	0.9311	0.0310	0.6796	0.7526	0.0540	0.6200	0.9535	0.0801	0.5168	0.8334
	Penicillium sp.	0.3001	0.0548	0.0222	0.1165	0.0113	0.0465	0.1848	0.0923	0.0172	0.0566	0.0967	0.0130
	Debaryomyces hansenii	0.5472	0.2323	0.0150	0.7438	0.2709	0.0670	0.6607	0.2425	0.0153	0.7543	0.3272	0.0405
	Debaryomyces sp.	0.0656	0.0283	0.0007	0.0920	0.0335	0.0071	0.0824	0.0287	0.0012	0.0923	0.0410	0.0057
**Sub-dominant taxa**	*Yamadzima* sp.	0.011	0.0006	0.0000	0.0001	0.0000	0.0000	0.0000	< 0.0001	0.0000	0.0002	0.0000	< 0.0001
	Yarrowia lipolytica	0.0000	< 0.0001	0.0000	< 0.0001	0.0000	0.0000	0.0001	0.0005	0.0001	0.0018	0.0002	0.0005
	Ascomycota	0.0000	0.0000	0.0003	0.0000	< 0.0001	0.0148	0.0000	0.0000	0.0000	0.0001	0.0004	0.0076
	Kluyveromyces sp.	< 0.0001	0.0000	< 0.0001	0.0018	0.0002	0.0007	0.0000	< 0.0001	< 0.0001	0.0000	0.0000	0.0000
	Mucor sp.	0.0007	< 0.0001	< 0.0001	0.0018	0.0002	0.0007	0.0000	0.0000	0.0000	0.0000	< 0.0001	0.0000
	Mucor circinelloides	0.0005	0.0003	0.0000	0.0014	< 0.0001	0.0006	0.0000	0.0000	0.0000	0.0000	0.0000	< 0.0001
**Some rare taxa**	*Yarrowia lipolytica*	0.0000	< 0.0001	0.0003	< 0.0001	0.0000	0.0000	0.0002	0.0005	0.0001	0.0018	0.0002	0.0005
	Saccharomycetales	0.0028	0.0005	0.0000	0.0014	0.0001	0.0003	0.0000	0.0000	0.0000	0.0000	0.0000	0.0000
	Onygenales	0.0000	0.0000	0.0000	0.0000	0.0000	0.0000	0.0000	0.0000	0.0000	0.0000	0.0000	0.0013
	Pichia fermentans	< 0.0001	0.0000	0.0003	0.0000	0.0000	0.0000	0.0000	< 0.0001	< 0.0001	0.0000	0.0000	0.0000

Table S5. Gross composition and physicochemical characteristics of P- and M-derived cheeses manufactured from raw or pasteurized milk.

	Raw			Pasteurized		
Variable	P	M	Significance	P	M	Significance
DM (%)	58.1 ± 0.18	59.9 ± 1.38		58.8 ± 2.29	59.2 ± 0.53	
Fat (%)	30.9 ± 0.58	30.3 ± 1.09		28.5 ± 0.87	29.3 ± 0.75	
pH	5.51 ± 0.02	5.49 ± 0.01		5.41 ± 0.01	5.44 ± 0.05	
Calcium (%)	0.69 ± 0.01	0.70 ± 0.03		0.67 ± 0.01	0.71 ± 0.03	
Phosphorus	0.52 ± 0.01	0.53 ± 0.02		0.53 ± 0.01	0.55 ± 0.03	
Chlorides (g of NaCl/100g)	2.15 ± 0.05	2.22 ± 0.15		2.05 ± 0.30	2.20 ± 0.05	
a_w core	0.95 ± 0.00	0.95 ± 0.01		0.95 ± 0.01	0.95 ± 0.00	
a_w rind	0.93 ± 0.00	0.91 ± 0.00		0.93 ± 0.01	0.91 ± 0.01	*

Legend: P, pasture-derived cheeses ; M, maize-derived cheeses; *, p-value ≤ 0.05.
